# What is an enhancer?

**DOI:** 10.1002/bies.202300044

**Published:** 2023-05-31

**Authors:** Henry Fabian Thomas, Christa Buecker

**Affiliations:** ^1^ Max Perutz Labs University of Vienna Vienna Austria

**Keywords:** enhancer, enhancer‐like element, facilitator element, polymerase II, transcriptional regulation

## Abstract

Tight control of the transcription process is essential for the correct spatial and temporal gene expression pattern during development and in homeostasis. Enhancers are at the core of correct transcriptional activation. The original definition of an enhancer is straightforward: a DNA sequence that activates transcription independent of orientation and direction. Dissection of numerous enhancer loci has shown that many enhancer‐like elements might not conform to the original definition, suggesting that enhancers and enhancer‐like elements might use multiple different mechanisms to contribute to transcriptional activation. Here, we review methodologies to identify enhancers and enhancer‐like elements and discuss pitfalls and consequences for our understanding of transcriptional regulation.

## Graphical abstract

Enhancers are defined as DNA sequences that can activate transcription independent of direction and distance to a promoter. However, many epigenomically identified putative enhancer elements might not conform to this original definition and contribute to transcriptional activation at the endogenous locus through a mechanism distinct from canonical enhancers.

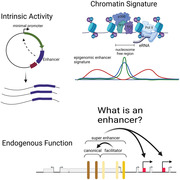

## INTRODUCTION

The human genome consists of over 3 billion bases, but only 2% contain the information for protein‐coding genes. A large fraction of the mammalian genome consists of repetitive elements such as transposable elements and endogenous retroviruses. Many of these elements have to be actively repressed to protect the organism.^1^ And finally, a sizable fraction of the genome is dedicated to organizing the 2 m of DNA within the nucleus and ensuring precise temporal and spatial gene expression patterns within an organism.^2^, ^3^, ^4^, ^5^ Each gene is active exactly when and where its function is required during development and tissue homeostasis. While many if not all steps of gene expression can be regulated, the very first step of gene expression—transcription of DNA into an mRNA molecule—is one of the most important regulatory events. During this process, RNA polymerase II is recruited to the promoter of a gene and initiates transcription with the help of the general transcription machinery and a set of regulatory co‐factors.^6^ However, promoters themselves often drive only basal levels of transcription. Additional *cis*‐regulatory elements such as enhancers are required for achieving appropriate levels of target gene expression.^2^, ^3^, ^4^, ^5^ The discovery of enhancers dates back more than 40 years ago with the realization that short sequences within the SV40 viral DNA can boost the expression from a minimal promoter independent of direction and distance to the promoter.^7^, ^8^ The source of enhancer activity are short DNA motifs that are recognized and bound by transcription factors (TFs). Upon binding to the DNA, these TFs recruit a cohort of co‐activators to indirectly activate transcription.^4^ Each cell type within an organism presents a specific gene expression signature that can be explained by cell type‐specific enhancer usage that in turn controls the specific gene expression patterns.^4^ Organization of the genome in 3D and enhancer function are closely interlinked: enhancers are often located kilobases away from their target gene and this distance has to be bridged somehow.^9^ With increasing linear distance, the activity of an enhancer on a target promoter drops,^10^, ^11^ but the constant extrusion of the genome through cohesin can lead to organizational structures that can favor enhancer‐promoter interaction or proximity even across very long distances.^12^, ^13^ But how close a promoter and an enhancer have to come together^14^, ^15^, ^16^ and how often this needs to happen for an active transcription event^11^, ^17^ is actively discussed. Mutations within enhancer sequences and genomic rearrangement can be the source of diseases such as congenital malformations or cancer.^18^

The original definition of an enhancer is very clear: a DNA sequence that activates transcription independent of direction and distance to a promoter.^7^ Many well‐characterized enhancer elements such as the Shh ZRS enhancer clearly fall under this definition.^19^, ^20^, ^21^ However, the classical strict definition has become softened through the usage of genomics technologies and high‐throughput identification of putative enhancer elements, and it is unclear how many elements categorized as enhancers fulfil the classical definition of an enhancer. Furthermore, it has become clear that some enhancer‐like elements might contribute to transcriptional activation through a mechanism clearly different from the classical enhancer definition.

## IDENTIFICATION OF ENHANCER SEQUENCES

### Combinatorial enhancer signature

**FIGURE 1:**
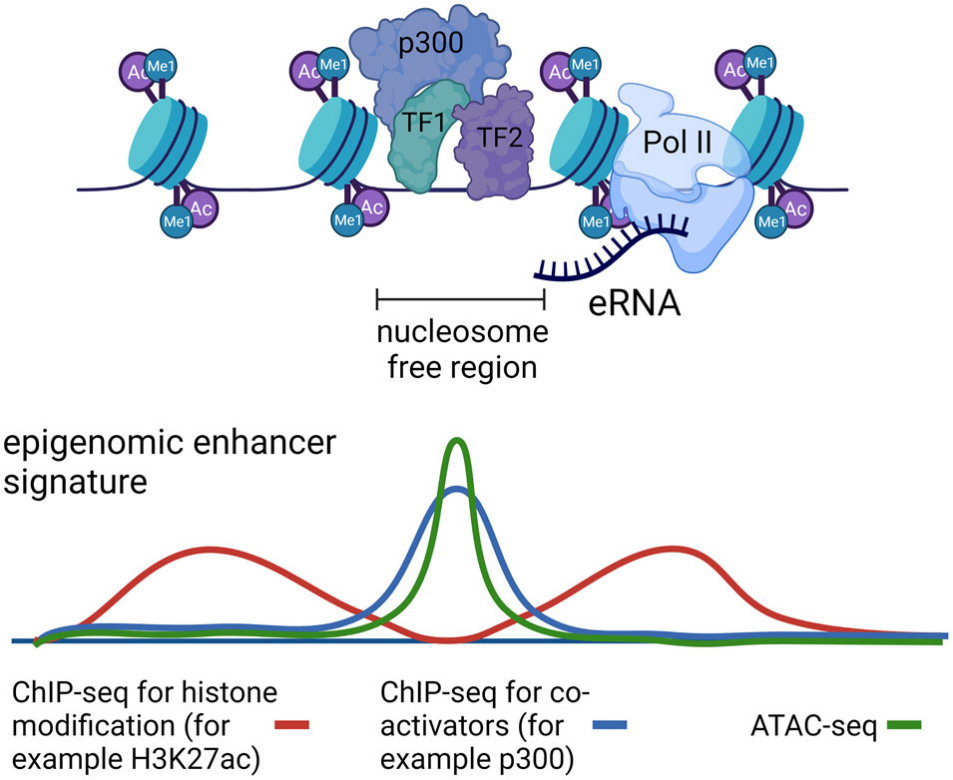
Epigenomic enhancer signature. At a typical enhancer, transcription factors (TFs) bind to their binding motifs and recruit co‐activators including chromatin remodelers or modifiers such as the acetyltransferase p300. Thereby, the nucleosomes are displaced from the core of the enhancer and lead to higher accessibility that can be measured by ATAC‐seq for example. The surrounding nucleosomes are often marked by typical modifications found at enhancers such as H3K27ac or H3K4me1. In addition, enhancers themselves are transcribed, so RNA polymerase II (Pol II) and short RNAs originating from enhancers called eRNAs can used for enhancer identification. Using either ChIP‐seq or ATAC‐seq, a typical epigenomic enhancer signature (bottom) can be used for identification for putative enhancer elements.

While originally enhancers have been identified based on their ability to activate gene expression, advances in next‐generation sequencing have enabled genome‐wide identification of putative enhancers based on their chromatin signature^4^, ^22^, ^23^ (Figure 1). Among the co‐activators recruited by enhancers are chromatin remodelers that can move or displace histones, thus increasing the accessibility of DNA and facilitating the binding of additional regulatory factors. Other co‐activators have catalytic activity and endow TFs, co‐factors, and histones with post‐translational modifications that are thought to be activating.^24^, ^25^ The combined action of these co‐activators results in a unique chromatin signature (Figure 1): while enhancers themselves have reduced histone density and increased accessibility, their surrounding nucleosomes are marked with the histone modifications H3K27ac and H3K4me1.^26^ This allows for the identification of an epigenomic enhancer signature with methods such as ATAC‐ and/or ChIP‐seq to map combinations of chromatin modification typically found at enhancers. However, there is an ongoing debate about which histone modifications are best suited to reflect true enhancers: while H3K27ac is widely used as the mark for an active enhancer, acetylation of multiple sites within the H2B N‐terminal tail might be better suited for enhancer prediction.^27^ As enhancers are usually transcribed and give rise to short, unstable transcripts referred to as eRNAs,^28^ mapping nascent transcription can also identify active enhancers.^29^, ^30^

In multiple recent studies, mapping of accessible chromatin has been used as the sole method to identify all potentially active *cis*‐regulatory elements within a cell type of interest. Specifically in single‐cell studies, the mapping of accessible chromatin is a great method to identify the regions of interest that could be important for cell identity,^31^, ^32^, ^33^, ^34^ and analysis of the TF binding motifs at these sites has allowed the reconstruction of the gene regulatory networks that safeguard the cell identity of interest.^33^ Using such a pipeline combined with novel deep learning approaches^33^ allows for the analysis of even rare cell types within a population that have not been accessible in the past due to low abundance and will open up deep views into gene regulation across a whole organism.

Therefore, the identification of putative enhancer elements based on epigenomic mapping allows scientists, specifically developmental biologists, to reconstruct the networks underlying gene expression changes during developmental transitions and in cell populations that were previously not tractable. However, not all putative enhancers identified based on their chromatin signature indeed affect gene expression at their endogenous locus^35^, ^36^, ^37^, ^38^ or are capable of activating a reporter gene in episomal enhancer assays^38^, ^39^, ^40^ or when integrated randomly into the genome,^38^ questioning their status as an enhancer.

### Massively parallel reporter assays (MPRAs)

**FIGURE 2:**
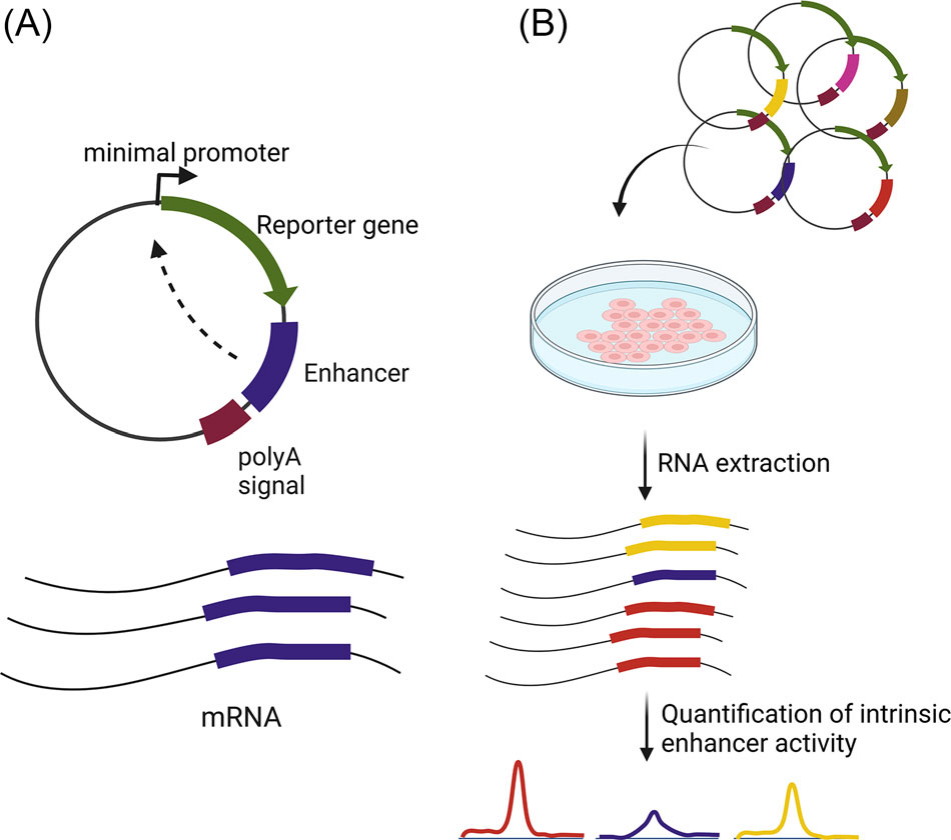
STARR‐seq as an example for a massively parallel reporter assays (MPRA). (A) When integrated into the transcription unit, elements with intrinsic enhancer activity can activate their own transcription and subsequently be identified through RNA‐Seq. In this example, the enhancer is cloned into the 3′UTR, between a reporter gene and the polyadenylation signal. (B) In a typical MPRA experiment, a library of sequences is incorporated into the MPRA vector and transferred into a population of cells. After RNA‐extraction and next generation sequencing, the elements with intrinsic enhancer activity can be identified and their activity quantified.

Alternative assays have been developed that allow high‐throughput identification of genomic fragments as enhancers based on their ability to activate a reporter gene. These assays are commonly referred to as massively parallel reporter assays (MPRAs, Figure 2).^41^ Libraries of DNA sequences that represent either the entire genome of a species,^39^, ^42^, ^43^ a smaller, preselected subset,^44^ or even artificially designed sequences^45^, ^46^ are integrated into a transiently transfected reporter plasmid or a reporter locus in the genome. Their ability to drive reporter gene expression from a minimal promoter can then be assayed. In a common strategy named STARR‐Seq, the reporter plasmids are designed in a way that enhancers drive transcription of their own sequence, which allows the mapping of highly active enhancers by RNA‐seq (Figure 2).^39^, ^42^, ^47^ MPRAs and lower‐throughput assays that test the sufficiency of a DNA sequence to activate transcription such as luciferase assays or lacZ transgenes are powerful tools,^48^, ^49^ as they directly assay the very definition of enhancer activity and have led to insights into enhancer biology. For example, MPRAs have been used to demonstrate remarkable enhancer‐promoter compatibility, that is, enhancers can activate some, but not other promoters.^50^ Specifically in the fly, housekeeping promoters are restricted to housekeeping enhancers and developmental promoters are predominantly activated and compatible with developmental enhancers.^50^ In mammals, it is actively debated whether such a clear separation exists.^51^, ^52^ Furthermore, it was demonstrated that not all enhancers identified through MPRAs activate transcription in the exact same way: for example, differential cofactor dependencies helped to identify several distinct classes of enhancers within the human genome. Some p53‐dependent enhancers do not require the mediator complex while other enhancers can still activate transcription in the absence of Brd4.^53^

The results above demonstrate the strength of directly assessing the intrinsic activity of an enhancer in a given cell type but also caution that the inability of a given sequence to activate the transcription of a single promoter does not necessarily exclude the intrinsic enhancer activity of that sequence when combined with a different promoter. In addition, the sufficiency of a DNA sequence to activate transcription might vary between episomal enhancer assays as compared to strategies that involve the integration of sequences into a reporter locus in the genome.^54^ Enhancer assays rely on a cut‐off to distinguish elements without intrinsic activity from elements with intrinsic activity, and in some cases have limited sensitivity and/or high variability between replicates, specifically when the whole genome is assayed. This can make it difficult to reliably characterize weak enhancers that might play an important role when combined with other weak enhancers at their endogenous locus^36^, ^37^, ^38^: many identified elements with typical enhancer chromatin signatures are not sufficient to activate gene expression but can do so when combined with other enhancers^36^, ^37^, ^38^ (see below for a more detailed description). Together, this cautions against over‐interpreting negative outcomes of MPRAs: there are many reasons why an individual sequence does not activate reporter gene expression in a given assay; this does not necessarily mean that the assayed DNA element cannot contribute to target gene expression in a different context. At the same time, not every enhancer sequence identified as active in MPRAs actually contributes to endogenous target gene expression: a non‐negligible fraction of enhancers that can activate a reporter gene in an ectopic context is not accessible at their endogenous locus in the genome, potentially due to active silencing at the chromatin level that is established during development.^42^, ^43^ In addition, MPRAs typically only assay a given cell type under defined conditions and will miss enhancers that become active in a slightly different developmental context or upon shifting environmental conditions.

### Deletion and silencing of endogenous enhancer elements

Finally, enhancers can be identified based on their ability to contribute to gene expression at their endogenous locus. Such studies typically involve the initial identification of a potential enhancer based on its chromatin signature, which is subsequently deleted from the genome to measure the effect on the surrounding genes.^38^, ^55^, ^56^, ^57^ As genome editing and identification of the target gene are not easily scalable, these studies are very low‐throughput and have only been performed at selected loci. Use of CRISPR interference (CRISPRi) can circumvent this limitation: combining a catalytically dead Cas9 (dCas9) fused to the KRAB repressor domain with a gRNA library allows induction of heterochromatin and silencing of many different candidate enhancers and identification of regulatory elements surrounding a gene of interest.^58^, ^59^ However, spreading of heterochromatin can lead to silencing of additional regulatory elements, and the efficiency of silencing depends on dCas9 recruitment and might be affected by which type of regulatory element is targeted.^58^, ^60^, ^61^ This complicates the quantitative analysis of CRISPRi experiments, especially when a gene of interest is regulated by several weak enhancers in close vicinity to each other. Furthermore, studies that delete or silence enhancers usually measure the requirement of DNA sequences for target gene expression rather than their sufficiency.^36^, ^55^ The redundancy of multiple enhancers activating a single gene can mask the contribution of a given sequence, which might only become obvious once additional elements at the locus are deleted.^35^, ^36^ In other cases, certain elements can boost target gene expression in the presence of other enhancers but fail to do so on their own.^12^, ^36^, ^37^ Based on reduced target gene expression in their absence, these elements might be classified as enhancers if no additional experiments were performed. However, as these elements are not sufficient to activate target gene expression, they do not fall under the original enhancer definition and it is up for debate whether these elements should be referred to as enhancers, or whether these “enhancer‐like” elements represent a class of their own that activates transcription via distinct mechanisms.

## “ENHANCER‐LIKE” ELEMENTS

Many genes are regulated not by a single enhancer, but by clusters of multiple enhancer elements. Some of these clusters are referred to as super‐enhancers,^62^ stretch enhancers,^63^ or locus control regions^64^ depending on their levels of co‐activator binding or for historical reasons. Their constituent elements can work together in a redundant,^36^ additive,^37^, ^55^ or super‐additive^36^, ^37^, ^38^, ^57^, ^65^ fashion. Interestingly, many individual elements of enhancer clusters qualify as “enhancer‐like” elements, as they either fail to activate their target gene in the absence of additional enhancers at the endogenous locus and/or have no activity in plasmid‐based reporter assays^12^, ^36^, ^37^, ^38^, ^56^

**FIGURE 3:**
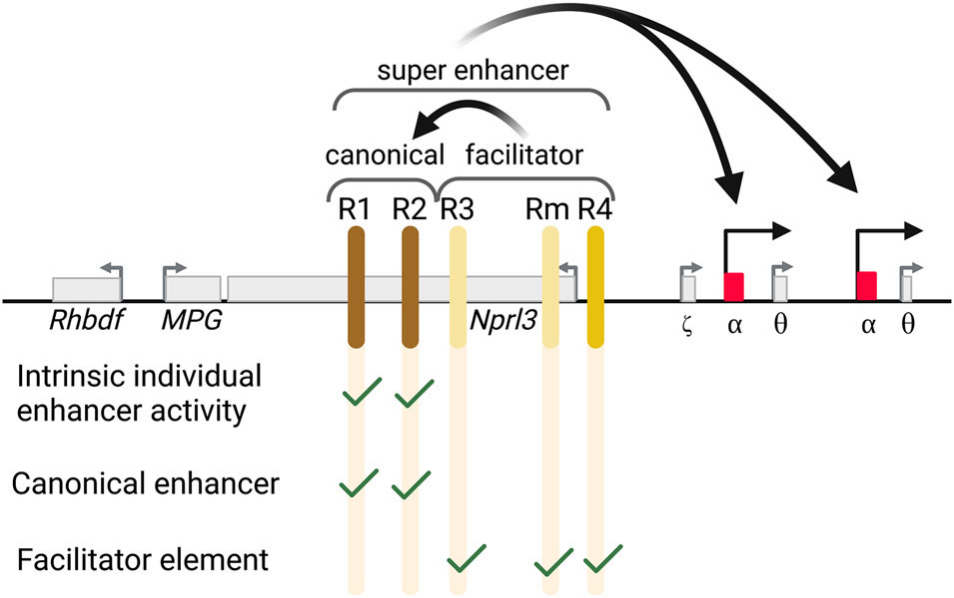
Canonical and facilitator elements cooperate to activate ɑ‐globin expression. The super‐enhancer at the ɑ‐globin locus consists of two canonical enhancer elements, that show strong intrinsic enhancer activity, and three facilitator elements, that have low or no intrinsic enhancer activity, but are needed within the context of the enhancer cluster to ensure proper activation of the duplicated ɑ‐globin gene promoters. The facilitator elements are redundant, so only deletion of all three elements leads to a strong reduction of the target gene expression. The mechanisms underlying facilitator action on canonical enhancers and target gene promoters is not understood yet.

Despite low intrinsic enhancer activity, deletion of these elements at their endogenous locus can lead to strongly reduced expression of the target genes. The molecular mechanisms underlying this behavior remain unclear and might vary from locus to locus. In some cases, multiple weak enhancers might work together in a super‐additive way, so that the deletion of a single element has a stronger effect than would be expected based on its intrinsic enhancer activity. At the *Fgf5* locus, for example, the individual enhancer elements present very low intrinsic enhancer activity in reporter assays, while individual deletion of elements can reduce the expression of the target gene *Fgf5* drastically, demonstrating the ability to contribute to gene expression despite low intrinsic activity.^38^ In other cases, “enhancer‐like” elements might deploy molecular mechanisms that are distinct from canonical enhancers to contribute to target gene expression. At the alpha‐globin locus, for example, the presence of so‐called “facilitator elements” is required for recruiting high levels of co‐activators to canonical enhancers at the locus and for establishing enhancer‐promoter contacts (Figure 3).^36^ Similarly, the dissection of the Sox2 control region (SCR), the main cluster of regulatory elements controlling the expression of the TF Sox2 in mouse embryonic stem cells, has identified elements that contribute to the regulation of Sox2 only when combined with other elements, but not alone.^37^ In other cases, “enhancer‐like” elements seem to mainly have an architectural role, either affecting contact frequencies within an entire TAD^12^ or establishing specific enhancer‐promoter contacts as in the case of “tethering elements”.^66^, ^67^ Similarly, orphan CpG islands (CGIs) are CGIs that are not in close vicinity to a promoter, they lack intrinsic enhancer activity but have been suggested to promote long‐range interactions of nearby poised enhancers with CpG‐rich developmental promoters.^68^

The elements described here are not sufficient to activate transcription yet play important roles in gene regulation of their target gene. They apply widespread mechanisms to affect their target gene, and additional classes of “enhancer‐like” elements that function in unique ways might await their discovery. These elements can be classified based on their mechanism of action; however, whether for example all “facilitator elements” or all “tethering elements” indeed apply the exact same mechanism remains to be dissected.

In the case of the globin‐locus, the three facilitator elements R3, Rm, and R4 behave slightly differently (Figure 3).^36^ Rm and R4 have a stronger effect when added to a locus only containing the canonical enhancers R1 and R2. Rm and R4 also display some level of redundancy, whereas the addition of R3 leads to a similar increase in target gene expression regardless of whether Rm or R4 are already present. The difference between R3 and Rm/R4 does not seem to be encoded in their underlying sequence though, as R3 can have as strong an effect as R4/Rm when it is introduced at the position of the R4 element. Thus, the local sequence context or chromosome conformation might play an important role. Alternatively, enhancer‐like elements might depend on the distance to the promoter, as has been described for canonical enhancers.^10^, ^11^

The elements described so far might mostly contribute to transcriptional activation by facilitating the interaction of classical enhancers with a target promoter across wider distances in 3D. Other elements that could also be classified as enhancer‐like elements might employ different mechanisms: they might be needed as activators of other enhancers,^57^ they might prepare a locus for activation at a completely different time point in development,^69^ or the transcription from the element might be needed for activation of a target locus.^70^ While not all these examples have been labeled as enhancers, they all show signatures that could classify them as enhancers under some circumstances. However, their mechanism of action is clearly different for each individual example.

All in all, these examples demonstrate that elements that could be labeled as “enhancer‐like” elements can behave quite differently and highlights the need to dissect the mechanism of each individual element in even more detail. At the same time, understanding the rules that define how a given element affects target gene expression will be helpful in the future, to distinguish canonical enhancers from the different classes of “enhancer‐like” elements. Furthermore, it is likely that some of the rules for enhancer‐like elements also apply to canonical enhancers but might be masked by their main transcriptional activation role.

## HOW TO DISTINGUISH CANONICAL ENHANCERS FROM FUNCTIONALLY DISTINCT ELEMENTS

The distinction of “enhancer‐like” elements from canonical enhancers implies a different underlying mechanism and is so far based on the lack of intrinsic enhancer activity of “enhancer‐like” elements. However, how to best measure intrinsic enhancer activity is not well defined. As discussed above, enhancer‐promoter compatibility and limited sensitivity of episomal or integrated enhancer assay can make it challenging to identify weak enhancers and claim a complete lack of any intrinsic enhancer activity. At the same time, the redundancy of several weak enhancers can mask the role of individual elements when they are deleted at the endogenous locus, and identifying enhancers just based on their chromatin signature can lead to the inclusion of many false positives.

We therefore strongly encourage combining a variety of different assays. Episomal enhancer assays—ideally performed with a set of different promoters including the endogenous one—are very useful to quickly understand whether a given element is sufficient to activate transcription. Deleting individual elements and their combinations at the endogenous locus is laborious and time‐consuming, but required to fully understand the interplay of all *cis*‐regulatory elements at a given locus.^36^, ^37^, ^55^ Fortunately, new tools are being developed that allow for the bottom‐up reconstruction and assembly of large genomic regions in yeast that can then be delivered into engineered mammalian cell lines and eventually mice to study many different combinations of individual enhancer deletions and reconfigurations.^36^, ^37^, ^71^ Analyzing resulting changes in TF and co‐activator recruitment, accessibility, as well as 3D genome conformation, is then needed to study the underlying mechanism by which each individual element contributes to the expression of the target gene. This will not only contribute to understanding how the different *cis*‐regulatory elements in the genome regulate transcription, but also allow us to distinguish weak canonical enhancers—that might escape detection based on limited sensitivity of the different assays—from “enhancer‐like” elements that are indeed functionally different from canonical enhancers.

## CONCLUSION

Here, we discussed the different methods that can be used for enhancer identification and their potential pitfalls. Newly identified, “enhancer‐like” elements transcend the original enhancer definition and play important roles in gene regulation despite low intrinsic enhancer activity. Careful combinations of different methods for enhancer identification need to be applied to ensure all regulatory elements at a given locus are identified and properly characterized. We envision future studies to characterize different classes of “enhancer‐like” elements, address their exact molecular mechanisms and identify features that distinguish them from canonical enhancers. This will greatly improve our understanding of how *cis*‐regulatory elements activate transcription, both in isolation and in combination with other elements.

## CONFLICT OF INTEREST STATEMENT

The author declares no conflict of interest.

## Data Availability

Data sharing not applicable to this article as no datasets were generated or analyzed for this review.
